# Disturbed Clockwork Resetting in Sharp-1 and Sharp-2 Single and Double Mutant Mice

**DOI:** 10.1371/journal.pone.0002762

**Published:** 2008-07-23

**Authors:** Moritz J. Rossner, Henrik Oster, Sven P. Wichert, Lisa Reinecke, Michael C. Wehr, Johannes Reinecke, Gregor Eichele, Reshma Taneja, Klaus-Armin Nave

**Affiliations:** 1 Max-Planck-Institute of Experimental Medicine, Göttingen, Germany; 2 Max Planck Institute of Biophysical Chemistry, Göttingen, Germany; 3 Mount Sinai School of Medicine, New York, New York, United States of America; Chiba University Center for Forensic Mental Health, Japan

## Abstract

**Background:**

The circadian system provides the basis to anticipate and cope with daily recurrent challenges to maintain the organisms' homeostasis. De-synchronization of circadian feedback oscillators in humans causes ‘jet lag’, likely contributes to sleep - , psychiatric - , metabolic disorders and even cancer. However, the molecular mechanisms leading to the disintegration of tissue-specific clocks are complex and not well understood.

**Methodology/Principal Findings:**

Based on their circadian expression and cell culture experiments, the basic Helix-Loop-Helix (bHLH) transcription factors SHARP-1(Dec2) and SHARP-2(Stra13/Dec1) were proposed as novel negative regulators of the molecular clock. To address their function *in vivo*, we generated *Sharp-1* and *Sharp-2* single and double mutant mice. Our experiments reveal critical roles for both factors in regulating period length, tissue-specific control of clock gene expression and entrainment to external cues. Light-pulse experiments and rapid delays of the light-dark cycle (experimental jet lag) unravel complementary functions for SHARP-1 and SHARP-2 in controlling activity phase resetting kinetics. Moreover, we show that SHARP-1 and 2 can serve dual functions as repressors and co-activators of mammalian clock gene expression in a context-specific manner. This correlates with increased amplitudes of *Per2* expression in the cortex and liver and a decrease in the suprachiasmatic nucleus (SCN) of double mutant mice.

**Conclusions/Significance:**

The existence of separate mechanisms regulating phase of entrainment, rhythm amplitude and period length has been postulated before. The differential effects of *Sharp*-deficiency on rhythmicity and behavioral re-entrainment, coupled to tissue-dependent regulatory functions, provide a new mechanistic basis to further understand the complex process of clock synchronizations.

## Introduction

Circadian rhythms in mammals are orchestrated by a pacemaker residing in the suprachiasmatic nucleus (SCN) of the hypothalamus [1]. The endogenous period length of this pacemaker deviates from the 24 h solar cycle and the clock therefore needs to be constantly reset to maintain synchrony with external time, a process termed entrainment. Light, transmitted to the SCN via the retino-hypothalamic tract, is the main entrainment stimulus (or *Zeitgeber*) for the core circadian oscillator [2]. Autonomous extra-SCN clocks in the forebrain and peripheral organs are synchronized via humoral and neuronal pathways and control the tissue and cell-type specific rhythmic expression of target genes [2], [3]. The oscillating expression of these clock-controlled genes is thought to provide the basis for an organism to anticipate and cope with daily recurrent challenges. Internal de-synchronization of peripheral and central oscillators causes ‘jet lag’ upon time-zone travelling and severely affects shift workers [4]. Genetic alterations in circadian genes have been shown to be linked to the familial advanced sleep phase syndrome (FASPS) [5], [6] and may contribute to psychiatric disorders [7]–[9] and even cancer [10], [11], highlighting the pivotal role of the circadian timing system in the maintenance of healthy body function.

Based on their rhythmic mRNA expression in the SCN and forebrain structures, the bHLH transcription factors SHARP-1(DEC2) and SHARP-2(DEC1) have been suggested as additional negative regulators of the mammalian clock [12]. They both bind to canonical E-Box *cis*-elements and repress CLOCK/BMAL1-mediated reporter gene expression in cell culture [12]. *Sharp-2* mRNA expression in the SCN was shown to be coupled to light stimulation [12] and neuronal activity in the cortex [13]. Analysis of *Sharp-2/Dec1* single mutant mice provided evidence for a role as a circadian output regulator in the periphery and in modulating phase of clock gene expression [14], [15].

## Results

**Figure 1:**
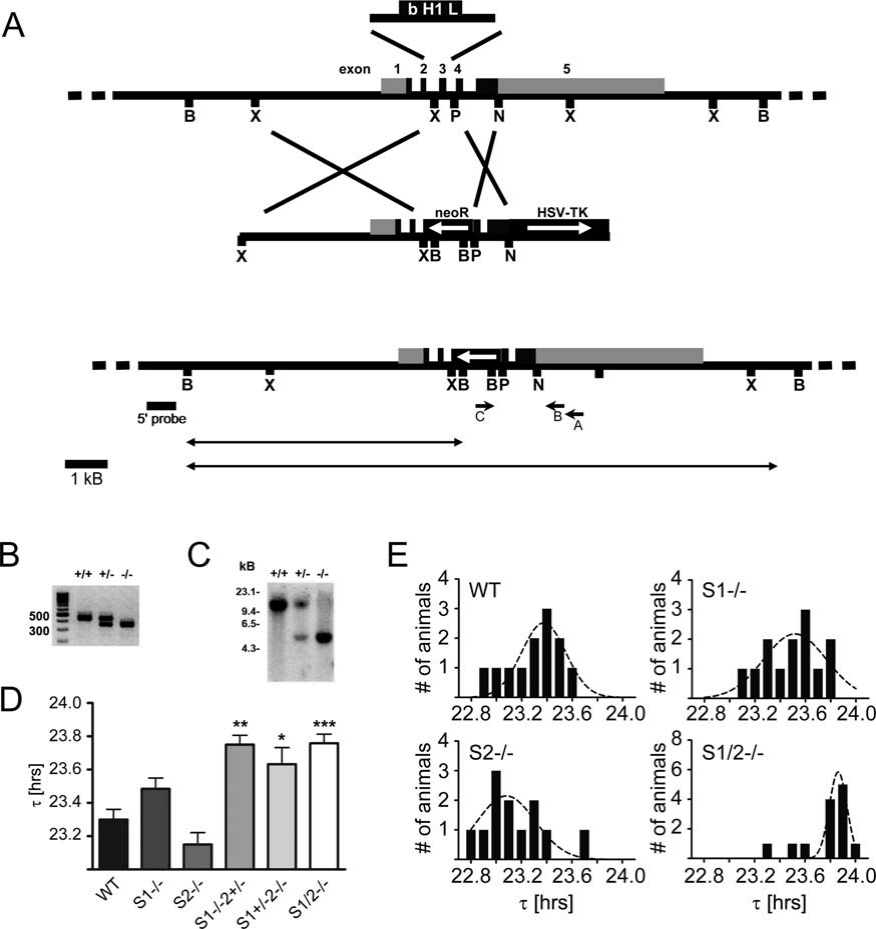
Gene dosage dependent alteration of free running periods in *Sharp* mutant mice. (a) Schematic drawing of the targeting strategy used to inactivate the *Sharp-1* gene in embryonic stem cells of the mouse. The *Sharp-1* gene is comprised of 5 exons (exon 1–5), coding sequences are depicted in dark grey, non-coding in light grey. The complete DNA binding domain encoded by exon 3 was replaced by a neomycin-resistance conferring cassette: For counter-selection the targeting vector was flanked with a thymidine kinase (HSV-tk) cassette. Locations of PCR primers used for ES cell screening are depicted as arrows below the targeted allele (A, B, C). The location of the DNA probe used for southern blot verification of successful targeting at the 5′ of the construct is indicated as bold line below the construct (5′ probe). Recognition sites for restriction enzymes are abbreviated as B (BamHI), X (XbaI), P (PstI) and N (NotI). (b) PCR analysis with the same genomic DNA as in (C) and primer pairs amplifying products corresponding to the WT (480 bp) and the mutated allele (327 bp). The PCR products span the region flanking the 3′ end of the targeting vector. (c) Southern Blot analysis of genomic DNA obtained from wild-type (+/+), heterozygous (+/−) and homozygous (−/−) *Sharp-1* mutant mice analyzed with radioactively labeled probe as indicated in (a). The genomic DNA has been digested with BamHI to verify correct targeting at the 5′ of the targeted locus. The detected bands at approximately 12 and 5 kb indicate the WT and mutated alleles, respectively. (d) Analysis of the free-running period length (τ) in DD. S1−/− and S2−/− mutants do not show a significantly altered τ compared to WT mice. Mice that lack three of four (S1−/− and S2+/−, S1+/− and S2−/−) or all wild-type *Sharp* alleles (S1/2−/−) display a significantly increased τ by 0.4–0.5 h. Values are 23.3 h±0.1 (WT), 23.5 h±0.1 (S1−/−), 23.2 h±0.1 (S2−/−), 23.8 h±0.1 (S1−/−S2+/−, p<0.01 = **), 23.6 h±0.1 (S1+/−S2−/−, p<0.05 = *) and 23.8 h±0.1 (S1/2−/−, p<0.001 = ***), respectively. (e) Bar graphs depicting the τ distribution of free running activity in DD for different *Sharp* single and double deficient mice. Curves show normalized distributions for each genotype. (Data are given as mean values ±SEM; n = 12 for WT, S1−/−, S2−/− and S1/2−/−; n = 6 for S1−/−S2+/− and S1+/− S2−/−).

In this study, we characterize the circadian system of mouse mutants lacking functional *Sharp-1* and/or *Sharp-2* genes. The targeted inactivation of *Sharp-2*/*Stra13* in mice has been described recently [16]. To inactivate *Sharp-1*, we replaced exon 3 coding for the DNA binding domain by a neomycin resistance cassette (Figure 1A). Genomic PCR and southern blot analysis proved successful targeting in ES cells and in mice (Figure 1B,C). RT-PCR with primers located in exon 1 and 4 revealed three aberrantly spliced mRNA species that might potentially lead to two different truncated protein fragments lacking any characterized functional domain (Supplementary Figure S1A–D). These truncated fragments were cloned in expression plasmids and appear to be non-functional (Figure S1E).

We assessed circadian clock function by monitoring wheel-running behavior of *Sharp* single and double mutants. Mice of all genotypes entrained normally to a 12 hr light/12 hr dark (LD) cycle (Figure S2 and data not shown). In constant darkness (DD), circadian activity rhythms remained stable without significant differences in free-running period lengths (τ) in single homozygous null mutants (Figure 1D,E). However, when *Sharp* gene dosage was further lowered, we observed a significant lengthening of τ (up to 0.5 h) in mice with only one or no functional *Sharp* allele left (Figure 1D,E and Figure S2) indicating a high degree of functional redundancy between both genes. The loss of one *Sharp* gene resulted in a broadened τ distribution indicating an elevated variability in pacemaker regulation while in the double mutants τ was consistently shifted towards higher values (Figure 1E).

**Figure 2:**
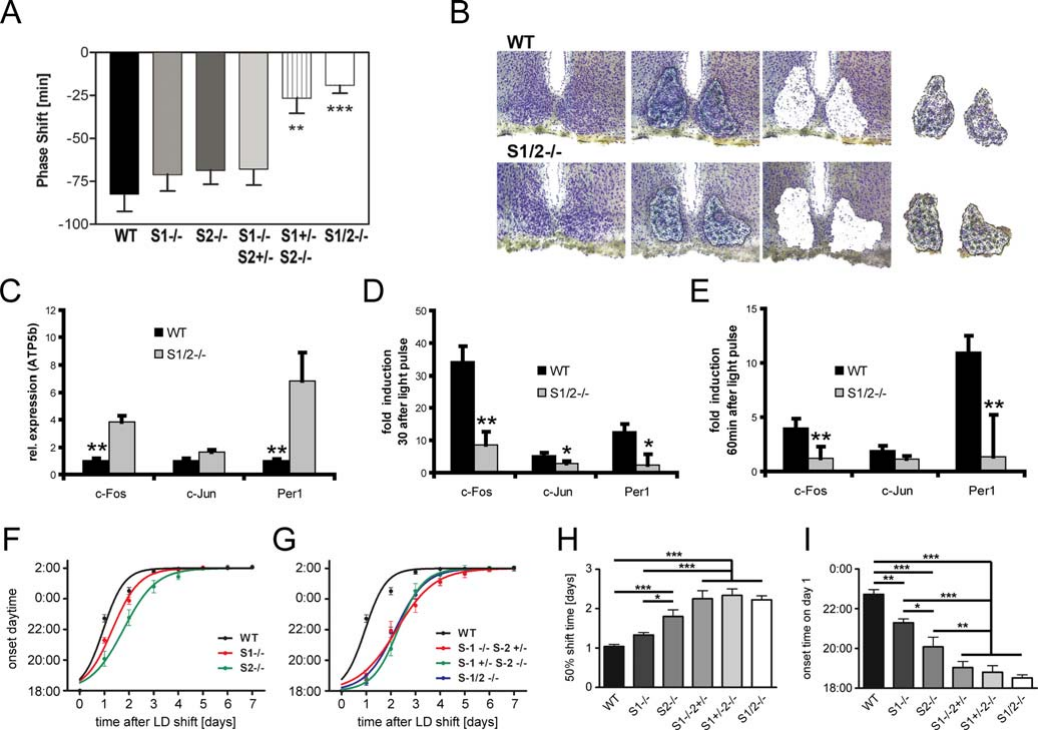
Disturbed phase shifting and clockwork resetting. (a) Acute activity phase shifts determined for WT and *Sharp* mutant mice upon a single 15 min light pulse given at ZT14 following the Aschoff type II protocol. S1−/− (71 min±7), S2−/− (69 min±6) and S1−/S2 +/− mutants (68 min±9) did not show a significant phase shift change compared to WT animals (82 min±10). S1+−/S2 −/− (27 min±9, p<0.01 = **) and S1−/S2 −/− (19 min±5, p<0.001 = ***) mutant mice showed a highly significant reduction in phase shift when compared to WT animals (Data are given as mean values ±SEM; n = 12 for WT, S1−/−, S2−/− and S1/2−/−; n = 6 for S1−/−S2+/− and S1+/− S2−/−). (b) Composite picture showing the steps of laser-mediated microdissection of representative WT and S1/2−/− thionin-stained coronal brain cryosections for collection of SCN tissue with high precision. Left panel, the thionin-staining allows for the detection of the SCN by light microscopy; 2^nd^ left panel, micrographs showing the regions selected for isolation; 3^rd^ left panel, sections after isolation; right panels, micrographs obtained from the caps that were used for isolation. (c–e) qRT-PCR data monitoring the relative *c-Fos*, *c-Jun* and *Per1* expression in the SCN of WT and Sharp mutant mice. Tissue was harvested at ZT14 from control animals without light pulse (c) and 30 min (d) as well as 60 min (d) after 15 min light exposure at ZT14. (c) At ZT14, *c-Fos* and *Per1* expression levels are significantly elevated and *c-Jun* concentration is slightly increased in the SCN harvested from S1/2−/− mice. (d,e) Photic induction of immediate early and *Per1* genes is highly reduced in S1/2−/− mice both 30 (D) and 60 min (E) after the light exposure (* = p<0.05, ** = p<0.01) (Data represent mean values ±SD, n = 3). (f–i) Re-entrainment of WT, S1−/−, S2−/−, S1−/−S2+−, S1+/−S2−/− and S1−/−S2−/− mutant mice to an 8 h delayed LD cycle, respectively. (f) *Sharp* single mutants display a significantly delayed resetting to the delayed LD cycle (ANOVA for interaction of factors ‘genotypes×time’ WT vs S1−/−: F(7,12.2) = 8.4, p<0.0005; WT vs. S2−/−: F(7,36.0) = 12.4, p<0.0005). *Sharp-2* mutants appear to adapt slower compared to *Sharp-1* mutants in this task. (g) The loss of additional *Sharp* alleles further delays the re-adaptation kinetics compared to the WT and single mutants in the 8 h phase delay paradigm. ANOVA for interaction of factors ‘genotypes×time’ WT vs S1−/−S2+−: F(7,50.2) = 23.6, p<0.0005; WT vs S1+/−S2−/− F(7,73.6) = 34.6, p<0.0005; S1−/−S2−/−: F(7,13.4) = 3.1, p<0.005). (h) Comparison of the half-maximal (50%) re-adaptation time in days of WT, S1−/−, S2−/−, S1−/−S2+−, S1+/−S2−/− and S1−/−S2−/− mutant mice reveals significant differences between WT and all other genotypes except *Sharp-1* mutants (* = p<0.05, ** = p<0.01, *** = p<0.001). (i) The onset time of running wheel activity at the first day after the LD shift reveals significant differences between WT and all genotypes. (Data are given as mean values ±SEM; n = 12 for WT, S1−/−, S2−/− and S1/2−/−; n = 6 for S1−/−S2+/− and S1+/− S2−/−; * = p<0.05, ** = p<0.01, *** = p<0.001).

Because *Sharp-2* expression in the SCN is rapidly activated after nocturnal light exposure, and remains elevated in the SCN in constant light conditions [12], we speculated that the *Sharps* could also be involved in the light synchronization of the SCN clock by light. Using a standard Aschoff type II paradigm, 15 min light pulses were given at *Zeitgeber* time (ZT)14, running-wheel activity was monitored after subsequent release into DD and the resulting phase shifts were calculated by comparison of activity onsets of light-pulsed animals and non-pulsed controls. Similar to what was seen for the period length in DD, *Sharp-1* deficiency did not significantly affect phase shifting behavior when compared to WT controls (Figure 2A). We observed, however, a markedly pronounced prolonged resetting kinetics (transition phase) in *Sharp-2*-deficient animals (data not shown). To further substantiate this finding, we tested WT and S2^−/−^ animals in an Aschoff type I setup applying a light pulse 2 hrs after activity onset under DD conditions (Circadian time (CT)14) when phase shift transients are more easily assessable. In this paradigm total phase shift amplitudes were again comparable between genotypes but S2^−/−^ mice needed consistently longer to complete the transition phase (Figure S3). We further reduced the gene dosage analyzing mice with only one functional *Sharp-2* (S1^−/−^S2^+/−^) or *Sharp-1* (S1^+/−^S2^−/−^) allele and homozygous double-deficient animals (S1/2^−/−^). S1^−/−^S2^+/−^ mice did not show a significant change in phase shifts (68±9 min) compared to WT animals (83±10 min) (Figure 2A). However, mice lacking both *Sharp-2* and one *Sharp-1* wild-type allele(s) as well as double mutants displayed marked reduction in phase shift responses (27±9 min and 19±5 min, respectively; Figure 2A). Although we again detected a functional redundancy of both *Sharp* genes in this paradigm, *Sharp-2* loss-of-function cannot be fully compensated for by only one *Sharp-1* wild-type allele. The amount and direction of photic activity phase shifts depends on the time of light exposure and involves differential molecular signaling mechanisms. To get a first idea about the circadian dependence of phase responses in *Sharp* mutant animals we also exposed all animals to light pulses at ZT22 (phase advancing) and ZT10 (no phase shift in wild-type mice). For both time points no significant differences were detected between wild-type and all *Sharp* single and double mutant animals (data not shown) indicating that the shape of the photic phase response curve is preserved in the absence of *Sharp-1* and/or *Sharp-2* and that the influence of both genes on clock resetting is restricted to phase delays.

The resetting of the SCN clock by light – and with it the activity phase - is thought to be the direct consequence of transcriptional activation of light-responsive genes in the SCN [2], [3], [17]. The prototypical immediate-early-genes *c-Fos* and *c-Jun* as well as *Per1* are known to be rapidly activated in the SCN after nocturnal light exposure [18]. We therefore microdissected SCN tissue from frozen coronal brain sections of mice that either received a 15 min light stimulus at ZT14 and were sacrificed 30 and 60 min later or were sacrificed under dim red light at ZT14 without prior light treatment (Figure 2B). The samples of three WT and three S1/2^−/−^ mice were pooled and subjected to quantitative RT-PCR analysis, monitoring *Per1*, *c-Fos* and *c-Jun* mRNA levels (Figure 2C–E). We observed elevated base levels of expression for all three genes already at ZT14 indicating alterations in the diurnal regulation of these genes in the absence of *SHARP-1* and *-2* (Figure 2C). In contrast, light induction of both *c-Fos* and *Per1* was significantly blunted both 30 (Figure 2D) and 60 min (Figure 2E) after light exposure in *Sharp* double mutant animals. These data correlate with the attenuated behavioral light responses observed in these mice and indicate a direct influence of the SHARP proteins on circadian gene regulation in the SCN.

We next investigated the impact of *Sharp*-deficiency on clockwork resetting linked to non-SCN oscillators [3], [4]. *Npas2*-deficient mice are characterized by blunted expression rhythms of *Per2* in the cerebral cortex [19] and adaptation to rapidly shifted LD cycles (*jet lag*) is accelerated when compared to WT controls [20]. Given the overlapping expression patterns of *Npas2* and *Sharp-1* and *-2* [12], [13], [19] and the proposed negative function of the SHARP proteins in the mammalian clock [12], we hypothesized that these animals should also show a related – but inverted – jet lag phenotype. Therefore, *Sharp*-deficient mice were subjected to a rapid 8 h delay of the LD cycle mimicking a flight from central Europe to Arizona. In WT animals, activity phase resetting to the new LD cycle occurred within 3 days after the shift (Figure 2F and Figure S4), in accordance with published data [20], [21]. In contrast, both *Sharp* single mutants displayed significantly slowed behavioral re-entrainment to the delayed LD cycle (two-way ANOVA for S1^−/−^, p<0.0005; S2^−/−^, p<0.0005) (Figure 2F). With respect to the resetting kinetics, *Sharp-1* mutants appeared to be less affected than *Sharp-2* mutants (Figure 2F). S1^−/−^S2^+/−^, S1^+/−^S2^−/−^ and double mutants (S1/2^−/−^) were also significantly delayed in their adaptation to a delayed LD cycle (S1^−/−^S2^+/−^, p<0.0005; S1^+/−^S2^−/−^, p<0.0005; S1^−/−^S2^−/−^, p<0.005) (Figure 2G–I and Figure S4). As judged by ANOVA, the additional loss of functional *Sharp-1* alleles, however, did not significantly alter the resetting kinetics when compared to S2^−/−^ mice, although the phase delay in particular during the first days was markedly reduced (Figure 2H–I). Thus, in contrast to the other phenotypes described so far, *Sharp-2* function seems to be essential for a rapid resetting to an 8 h delayed LD cycle and cannot be compensated for by *Sharp-1*. These effects appear to be tied to tissue-dependent function of SHARP proteins, e.g. in the forebrain clock.

**Figure 3:**
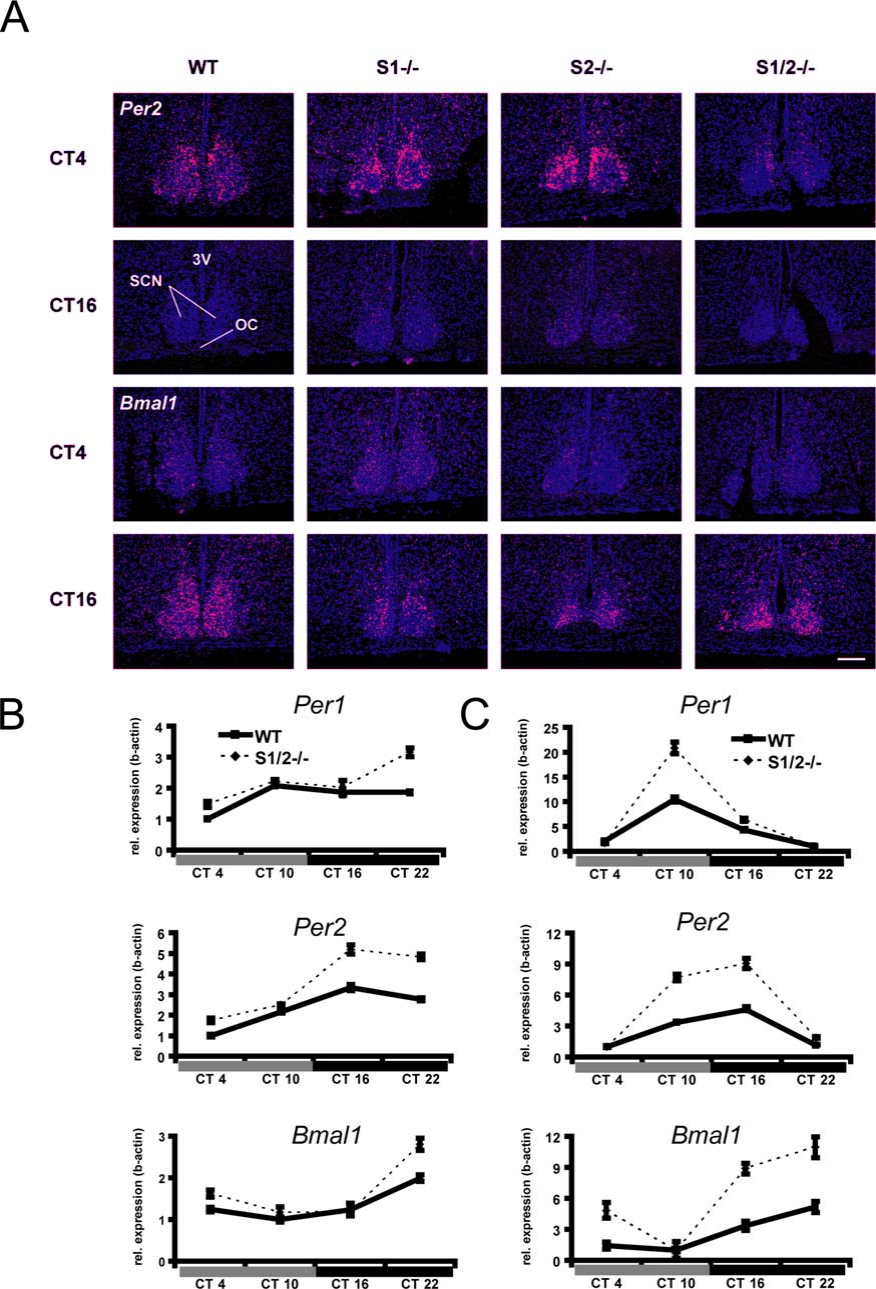
Altered clock gene expression in the SCN, cortex and liver. (a) In situ hybridization at the level of the SCN using ^35^S-labeled riboprobes directed against *Per2* and *Bmal1* mRNAs. In WT, S1−/− and S2−/−, *Per2* mRNA expression in the SCN is strongly elevated at CT4 compared to CT16 reflecting a clock-controlled rhythm of expression. In S1/2−/− mice, the increase of *Per2* mRNAs expression during the subjective day is blunted. *Bmal1* expression is elevated at CT16 in all genotypes (3V, third ventricle; SCN, suprachiasmatic nucleus; OC, optic chiasm). Scale bar = 200 µm. (b,c) Relative gene expression changes during four consecutive circadian timepoints (CT4, 10, 16 and 22) in wild-type (WT) and *Sharp-1/-2* double mutant (S1/2−/−) cerebral cortex (b) and liver (c) revealed by qRT-PCR. The amplitude of the circadian regulated gene expression of *Per1*, *Per2* and *Bmal1* is significantly enhanced during the first day of DD comparing WT and S1/2−/− mice (p<0.05 for *Per1* in the cortex, all other comparisons p<0.01 with 2-way ANOVA and the factors ‘genotypes×time’). (Data represent mean values ±SD, n = 3 per genotype).

Based on the differential effects of *Sharp*-deficiency on rhythmicity (Figure 1) and behavioral re-entrainment (Figure 2), we analyzed clock gene expression rhythms in different tissues of WT and *Sharp*-deficient mice in DD. In concordance with previous reports [22], *in situ* hybridizations using ^35^S-UTP-labelled RNA probes at two different time points of the circadian cycle revealed anti-phasic *Per2* and *Bmal1* expression in the SCN of WT mice (Figure 3A). This rhythm of expression was largely preserved in both *Sharp-1* and *Sharp-2* single mutant mice (Figure 3A). However, correlating with the lengthened τ in the double mutant mice, *Per2* expression rhythms in S1/2^−/−^ animals appeared severely blunted with highly reduced mRNA levels observed at CT4 while *Bmal1* expression appeared normal (Figure 3A). This is supported by similar findings from other circadian clock mutant mice linking dampened *Per* gene expression rhythms to a lengthened clock period [23], [24], but contradict previous *in vitro* studies suggesting that SHARP-1/-2 act exclusively as transcriptional suppressors of clock genes (including *Per2*) [12], [15]. As tissue-specific effects have been described for other circadian clock gene mutations [23], [25], we compared clock gene expression rhythms of *Per1*, *Per2* and *Bmal1* in extra-SCN tissues in WT and S1/2^−/−^ mice by quantitative RT-PCR. Rhythmic expression of these genes during the first day in DD was apparent in WT mice and *Sharp* mutants in both the somato-sensory cortex (Figure 3B) and liver (Figure 3C). Baseline levels and amplitudes of *Per1*, *Per2* and *Bmal1* mRNAs, however, were found to be elevated in cortex and liver of S1/2^−/−^ mice, albeit with marked quantitative differences between both tissues (Figure 3B,C).

**Figure 4:**
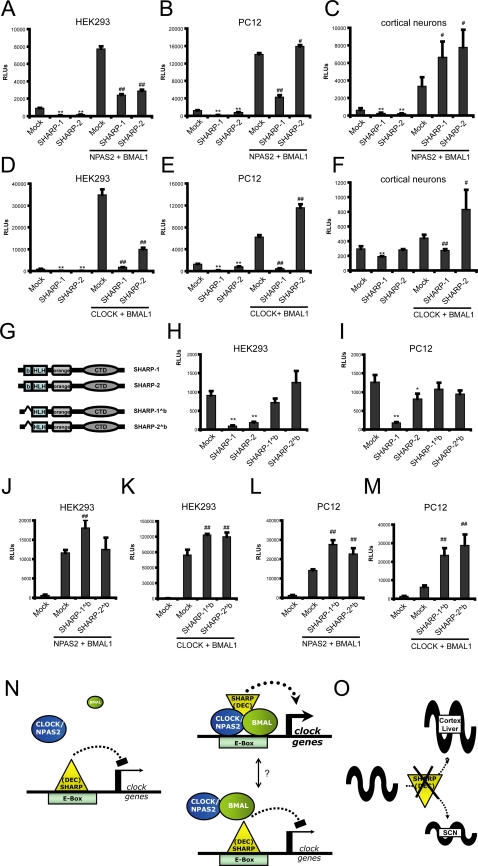
Context-dependent repressor or co-activator functions of SHARP-1 and -2. (a–f) Reporter gene assays using a firefly luciferase reporter construct driven by a herpes simplex thymidine-kinase (TK) minimal promoter and three upstream clustered CACGTG E-Box elements and BMAL1, NPAS2 and SHARP-1 or SHARP-2 expression plasmids performed in HEK293 cells (a,d), PC12 cells (b,e) and primary cultured mouse cortical neurons (c,f) as indicated. (a,d) SHARP-1 and -2 repress basal and NPAS2/BMAL1 (a) and CLOCK/BMAL1 (d) enhanced promoter activity in HEK293 cells. Co-transfection of NPAS2 or CLOCK with BMAL1 encoding plasmids activates the reporter gene more than 8- and 30-fold, respectively. SHARP-1 and -2 efficiently repress NPAS2/BMAL1 and CLOCK/BMAL1 mediated reporter gene activity. (b,e) SHARP-1 represses basal and NPAS2/BMAL1 (b) and CLOCK/BMAL1 (e) enhanced promoter activity in PC12 cells whereas co-transfection of SHARP-2 encoding plasmids has no effect on NPAS2/BMAL1 mediated luciferase activation (b) but further stimulates CLOCK/BMAL1 activities by almost two-fold (e). (c,f) Reporter gene assays performed in primary cultured neurons. (c) SHARP-1 and -2 repress basal promoter activity in reporter gene assays performed in primary cultured neurons, but co-activate NPAS2/BMAL1 mediated reporter activation. Co-transfection of both BMAL1 and NPAS2 leads to an increase of luciferase activity by approximately 6-fold and SHARP-1 and -2 further enhance this activation by a factor of two to at least 12-fold over basal promoter activity. (f) SHARP-1 represses CLOCK/BMAL1 activity and SHARP-2 stimulates CLOCK/BMAL1 activity by more than two-fold. (g) Domain structures of full-length SHARP-1 and -2 and deletion mutants lacking the DNA binding basic domain, SHARP-1ˆb and SHARP-2ˆb. B, basic domain; HLH, Helix-Loop-Helix domain; orange, orange domain; CTD, C-terminal domain. (h–m) Reporter gene assays using a firefly luciferase reporter construct driven by a herpes simplex thymidine-kinase (TK) minimal promoter and three upstream clustered CACGTG E-Box elements and BMAL1, NPAS2, CLOCK and SHARP-1ˆb and SHARP-2ˆb expression plasmids performed in HEK293 cells (h,j,k), PC12 cells (i,l,m) as indicated. (h,i) Full-length SHARP-1 and -2 repress E-Box mediated reporter gene activities in HEK293 (h) and PC12 cells (i) whereas mutants lacking the basic domains (SHARP-1ˆb and SHARP-2ˆb) have no significant repressive effect. (j,m) SHARP-1 and -2 mutants lacking the DNA binding basic domain (SHARP-1ˆb and SHARP-2ˆb) are co-activators of BMAL1/NPAS2 (j,l) and BMAL1/CLOCK (k,m) mediated transcription in HEK293 (j,k) and PC12 (l,m) cells. * and ** indicate p<0.05 and p<0.01 when comparing SHARP-1 and -2 functions on basal promoter activities (Mock). # and ## indicate p<0.05 and p<0.01 when comparing SHARP-1 and -2 functions on NPAS2/BMAL1 and CLOCK/BMAL1 mediated reporter gene activations. RLUs = refererence light units. (Data represent mean values ±SD, n = 6 replicates). (g,h) Schematic model for the context-specific SHARP-1 and -2 functions regulating the amplitude of clock gene expression. (g) When BMAL1 levels are low, SHARP proteins likely act as repressors by direct E-Box binding independent of CLOCK or NPAS2 (left panel). In the presence of BMAL1 and CLOCK or NPAS2, the SHARP proteins can either repress or activate E-Box mediated clock gene transcription. The mechanism regulating the context-dependent switch between repression versus activation remains to be identified. (h) In the cortex and liver, loss of SHARP function leads to an increased amplitude of clock gene expression, whereas the expression of the clock controlled gene (e.g. *Per2*) is dampened in the suprachiasmatic nucleus (SCN) of SHARP-1 and -2 double mutant mice.

In light of our data, we hypothesized that the SHARPs could potentially serve a dual function as activators (e.g. in the SCN) and as repressors of E-box-mediated transcription (e.g. in cortex or liver), extending the ‘repressor-only’ model derived from previous studies [12], [26], [27]. Likewise, the *Drosophila* homologue of SHARP-1 and -2, CWO, may repress or activate clock gene transcription under certain conditions [28], [29]. Given that the composition of the clock machinery varies between different tissues [3] and that SHARP-1 and -2 interact with the circadian transcriptional oscillator via E-box regulation [12], [15], [26], [27], we asked whether SHARP action might depend on the composition of the E-box activator complex. In the forebrain, CLOCK is functionally replaced by another bHLH-PAS factor, NPAS2 [19]. We thus used luciferase reporter gene assays to assess the effect of SHARP-1 and SHARP-2 on CLOCK/BMAL1 and NPAS2/BMAL1 activities in different heterologous cell lines and primary cultured cortical neurons. In accordance with published data [12], [15], [26], [27]}, SHARP-1 and -2 efficiently repressed transcription of a luciferase reporter driven by canonical CACGTG E-Box enhancer elements in HEK293 cells (Figure 4A,D and Figure S5A). In the presence of either BMAL1, NPAS2 or CLOCK, similar levels of repression were observed for both SHARP-1 and -2 (Figure S5A). Co-transfection of either NPAS2/BMAL1 and CLOCK/BMAL1 expression constructs substantially induced reporter gene transcription (>7-fold for NPAS2/BMAL1 and >30-fold by CLOCK/BMAL1) (Figure 4A,D) and both SHARP-1 and -2 efficiently repressed NPAS2/BMAL1 and CLOCK/BMAL1 activities several fold (Figure 4A,D). Similar effects were also obtained in CHO cells (Figure S6), in which we also compared the levels of SHARP-1 and -2 mediated repression with those of CRY1 and -2, which are the most potent repressors of clock-gene transcription [30]. The repressive effect of SHARP-2 was comparable to that of CRY-1/-2 and SHARP-1 was even more potent than CRY-1/-2 (Figure S6). These data confirm the current model that SHARP-1 and -2 act as transcriptional repressors. Next, we performed similar reporter gene assays in PC12 cells (Figure 4B,E and Figure S5B) and primary cultured cortical neurons (Figure 4C,F and Figure S5C). In both cell types, SHARP-1 and -2 showed a significant repression of the basal reporter gene activity between 2- and 6-fold and when either BMAL1, NPAS2 or CLOCK expression plasmids were co-transfected (Figure S5B,C). Upon co-transfection of NPAS2/BMAL1 or CLOCK/BMAL1, however, SHARP-1 and -2 behaved both as repressors and co-activators of transcription. In PC12 cells, SHARP-1 suppressed both NPAS2/BMAL1 and CLOCK/BMAL mediated activation, whereas SHARP-2 did not (Figure 4B,E). However, we obtained a robust, almost two-fold increase in luciferase activities when CLOCK/BMAL and SHARP-2 were co-expressed (Figure 4E). Moreover, in primary cultured cortical neurons, SHARP-1 and -2 robustly co-activated NPAS2/BMAL1 transcription by at least two-fold (Figure 4C). CLOCK/BMAL1 transcription, however, was repressed by SHARP-1 and enhanced by SHARP-2 (Figure 4F).

To follow up on the dual mode of SHARP protein function in more detail, we used mammalian two-hybrid assays (Figure S7) and monitored the subcellular localization of NPAS2 upon co-transfection with either BMAL1, SHARP-1 or SHARP-2 encoding expression plasmids (Figure S8). These and previous experiments (Figure 4 and Figure S5) reveal that SHARP-1 and -2 most likely do not interact via the HLH domain with BMAL1, NPAS2 and CLOCK to either repress or co-activate as heterodimeric complexes clock gene expression in an E-Box dependent manner. Quantification of the effects of SHARP-1 and -2 reporter assays show that the co-activator function is dependent on BMAL1/NPAS2 or BMAL1/CLOCK complexes but is E-Box independent (Figure S9). We thus generated SHARP-1 and -2 constructs lacking the DNA binding domains (SHARP-1ˆb and SHARP-2ˆb) and assessed their function in reporter gene assays. In HEK293 and PC12 cells, the SHARP-1ˆb and SHARP-2ˆb proteins could not repress E-Box mediated reporter gene transcription when transfected seperately (Figure 4H,I). However, SHARP-1ˆb and SHARP-2ˆb further stimulated the NPAS2/BMAL1 or CLOCK/BMAL1 driven reporter gene expression (up to 4-fold in PC12 cells) (Figure 4L–M), with one exception. In HEK293 cells, SHARP-2ˆb did not enhance NPAS2/BMAL1 activities (Figure 4J) but, unlike SHARP-1 (Figure 4A), did not display any repressive effect.

## Discussion

Taken together, our data show that SHARP-1 and -2 act both as E-Box binding dependent transcriptional repressors and as co-activators of NPAS2-CLOCK/BMAL1 in reporter gene assays which correlates with the differential *Per2* gene regulation in vivo. In the absence of functional NPAS2-CLOCK/BMAL1 heterodimers, SHARP-1 and -2 have an inhibitory function, which is in accordance with published data [12], [26], [27]. However, in the presence of functional NPAS2-CLOCK/BMAL1 heterodimers, SHARP-1 and -2 likely act either as directly E-Box binding repressors or as co-activators of transcription depending on the context and the nature of E-Box binding complexes (Figure 4N) and may thus regulate the amplitude of clock gene expression in different tissues (Figure 4O).

The activity of SHARP-1 and -2 on clock gene transcription is thus principally different to that of CRY1 and CRY2 [30]. Deletion of both *Cry1* and *Cry2* alleles in mice leads to a loss of rhythmicity in running wheel activity in DD and both genes are therefore essential negative components of the core clock of the SCN [31]. In contrast to the *Cry* genes, our analyses show that *SHARP-1* and *-2* are essentially involved in the fine-tuning of circadian functions such as period control, clockwork resetting and entrainment in the SCN as well as in clocks operating in other tissues such as the forebrain or liver. A similar effect on regulation of clock gene amplitude has been described for *Drosophila* mutants lacking *cwo* [28], [29], [32], [33].

Our *in vivo* and cell culture analyses suggest a tissue/cell type-dependent function of SHARP proteins in the circadian clock. *Sharp-1* and *Sharp-2* are expressed in the SCN as well as in other tissues and brain regions where an extensive overlap with NPAS2 expression domains is apparent [12], [13], [19]. The phenotypes of *Sharp* mutants in the jet lag paradigm and activity periods most likely represent the cumulative net effect of altered SHARP-1 and/or SHARP-2 functions integrated from different circadian oscillators, including the SCN pacemaker, where CLOCK dominates, as well as other CNS timers like the forebrain clock, where NPAS2 is essential. This tissue-specific clockwork composition appears as the key to SHARP functions. Further molecular and behavioral studies are required to dissect the cellular and molecular mechanisms that may be modulated by SHARP-1/2 in different cell types or tissues. The mechanism that determines the context-dependent switch between a repressing versus activating mode of SHARP function remains to be determined. However, our data with SHARP constructs lacking only the basic domain implies that this may occur post-translationally regulating the E-Box binding. The existence of separate mechanisms regulating phase of entrainment, rhythm amplitude and period length of the circadian system has been postulated before [6], [34]. The differential effects of *Sharp*-deficiency on free-running rhythmicity and behavioral re-entrainment, coupled to tissue-dependent SHARP regulatory functions, provide a new mechanistic basis to support this concept. An improved mechanistic insight of clock de-synchronization processes is likely to be relevant for several human diseases with altered ‘clocks’.

## Materials and Methods

### Mutant Mice

Targeting of the *Sharp*-1 locus was performed by standard techniques for homologous recombination in mouse embryonic stem cells. Heterozygous *Sharp-1* and *Sharp-2* mice, which had been back-crossed to C57BL6/J for more than ten generations, were mated to obtain double-heterozygous mutants and crossed to obtain double null-mutant mice. None of the *Sharp*-mutant strains displayed overt behavioral abnormalities. The genotypes analyzed in this study were WT (wild-type mice), S1^−/−^ (homozygous *Sharp-1* mutants), S2^−/−^ (homozygous *Sharp-2* mutants), S1^−/−^S2^+/−^ (homozygous *Sharp-1* and heterozygous *Sharp-2* mutants), S1^+/−^S2^−/−^ (heterozygous *Sharp-1* and homozygous *Sharp-2* mutants) and S1/2^−/−^ (homozygous *Sharp-1/2* double mutants).

### Gene targeting and homologous recombination in mouse ES cells

Gene targeting in mouse embryonic stem cells was performed as already described [35]. Briefly, a λ mouse 129Sv genomic library (Stratagene, La Jolla, CA) was screened with a full-length rat SHARP-1 cDNA, and several clones containing the five-exon gene [13], including at least 3 kb of 5′- and 5 kb of 3′-flanking sequences each, were isolated. A gene-targeting vector for positive-negative selection was constructed by subcloning the neomycin resistance (neoR) gene (modified by PCR from pMCneo; Stratagene) as a XbaI-PstI fragment into a linearized vector pKS (Stratagene). A XbaI fragment inluding 4 kb 5′ upstream and parts of intron 2 was placed 3′ of the neoR cassette. A NotI-PstI fragment containing 3′ sequences of the SHARP-1 gene was placed 5′ to the neoR cassette. For negative selection, the HSV-tk gene (modified by PCR from pMC1TK; Stratagene) was inserted into the NotI-SacII linearized construct. The targeting construct lacked the DNA binding domain located in exon 3 but transcription of the mutant gene could be monitored at the RNA level using a sense neoR specific probe. R1 mouse embryonic stem (ES) cells were cultured on mitomycin-treated primary mouse embryonic fibroblasts (feeders). For electroporation (240 V, 500 µF; Gene Pulser; BioRad, Munich, Germany), ∼10^7^ ES cells were suspended in 0.8 ml of PBS containing 50 µg of the linearized (ScaI) targeting construct. Transfected cells were cultured on gelatinized dishes (Falcon) in the presence of 10^3^ U/ml leukemia inhibitory factor (GIBCO), and selection with 300 µg/ml G418 (GIBCO) and 2 µM ganciclovir (Syntex) was started after 48 and 72 hr, respectively. Between 8 and 10 d after electroporation, double-resistant colonies were picked and trypsinized. Approximately one third of each colony was plated onto feeder cells. The remaining cells of eight clones each were pooled, suspended in 50 µl of water, heat-lysed at 95°C for 10 min, and digested with 10 µg of proteinase K at 55°C for 30 min.

To identify homologous recombinants, we used aliquots of the pooled lysates to PCR amplify a 1.2 kb genomic fragment. Two primers located in exon 5 3′ of the targeting construct at position A (S1-KOA, 5′-TGTCGGTGTCCGTGTCGTGT-3′) and position B (S1-KOB, 5′-AGACTTCCGTGGCTCTTAGAAC-3′) in combination with one primer located in position C of the targeting construct (corresponding to the 5′ end of the neoR cassette) (neo6, 5′-GCAATCCATCTTGTTCAATGGC-3′) were used (locations depicted in Figure 1A). In a 50 µl reaction, ‘nested’ PCRs were performed with denaturation at 94°C (30 sec), annealing at 56°C (45 sec), and extension at 72°C (60 sec) for 19 cycles (using S1-KOA and neo6) followed by 35 cycles with primers S1-KOB and neo6. Amplification products were visualized on ethidium bromide-stained gels and verified by Southern blot analysis. Correct targeting was also confirmed by Southern blotting of ES cell DNA (10 µg), hybridized to a 5′ genomic probe (see location in Figure 1A). Microinjection of selected ES cells into C57Bl/6J blastocysts was performed by standard procedures. Highly chimeric males were crossed to C57Bl/6J females.

For routine analysis, offspring genotype was determined by PCR on tail-biopsy DNA with primers S1-s (5′-ACCTACAAGTTACCGCACAG-3′), S1-as (5′-TTTCTCCAAATGCCCCAGTG-3′), and neo6. The wild-type allele yielded in a single 420 bp product amplified with S1-s and S1-as and the mutant allele in a single product of 350 bp amplified with neo6 and S1-as.

### SHARP-1 and -2 mutant mice

The experiments were performed with cohorts of male mice aged 3–6 months, respectively. Wild-type (WT) and *Sharp-1* and *-2* mutant mice were obtained from offspring of double heterozygous breeding pairs. Parental single heterozygous mice were independently backcrossed to C57Bl/6J for more than ten generations. All experimental animals were housed in the same ventilated sound-attenuated rooms under a 12 h light/12 h dark schedule at an ambient temperature of 21°C with food and water available *ad libitum*. All experiments were performed blinded for genotype and were conducted in accordance with NIH principles of laboratory animal care and were approved by the Government of Lower Saxony, Germany.

### Locomotor activity monitoring and circadian phenotype analysis

Mice housing and handling were performed as described [36], [37]. Activity data were recorded and evaluated using the ClockLab data collection and analysis software (Actimetrics, IL). Prior to all experiments, animals were entrained to a 12 hrs light: 12 hrs dark (LD) cycle for at least 2 weeks. For LD-DD transitions, lights were turned off at the end of the last light phase and not turned back on the next day. Total activity in LD and constant darkness (DD) and relative light phase activity in LD were assessed over a time of 5 consecutive days. Rhythmicity and endogenous period length (τ) in DD were analyzed by χ^2^ periodogram analysis over an interval of 5 days. For LD phase-shifting experiments (experimental jet lag) animals were, after 2 weeks of stable entrainment to a first LD cycle, subjected to an elongated night phase followed by an 8 hrs backward shifted LD cycle. Adaptation time was calculated by comparing activity phase onset times on the days after the LD shift as determined by the ClockLab software.

To analyze acute phase shifting by single nocturnal light pulses both Aschoff type I and II paradigms were used [38]. For type I individual period lengths and activity onsets were determined for each animal in DD and 15 min light pulses (400 lx) applied at two circadian hours (τ/12) after activity onset (CT14) before releasing the animals back into DD. Phase shifts were calculated as the time difference between two regression lines through the activity onsets before and after the pulse on the day of the light exposure. The first days after the light pulse were excluded from the analysis and classified as transition time. For the type II protocol animals were entrained to an LD cycle, 15 min light pulses were applied two (ZT14) or 10 hrs (ZT22) after “lights off” and animals released into DD. Phase shifts were determined as the difference between two regression lines through onsets before and after the pulse on the day of the light exposure as compared to animals of the same genotype which were released into DD without extra light treatment. Again, the first days in DD were not used for the calculation because animals were thought to be in transition between both states.

### Tissue Sampling for RT-PCR

For qRT-PCR, frontal cortex (bregma 0 to −2 mm) was isolated using a ‘rodent brain matrix’ 1 mm coronal slicer (ASI Instruments, Warren, MI) and pooled from three mice of each genotype. Microdissection of SCN tissue was performed according to published protocols [39]. In brief, brains from WT and S1/2−/− mice were harvested 30 and 60 min after 15 min light pulse given at ZT14 and were frozen on dry ice (n = 3 for each genotype and timepoint). Cryosections (15 µm) were obtained at the level of the SCN and sections were dip-stained with thionin (0.01% in 5% acetic acid). This protocol was shown not to affect RNA integrity [39]. The SCN was identified by the Nissl-morphology and pure SCN tissue was collected by laser-capture microdissection (Molecular Devices, CA; Veritas system) from at least 10 subsequent sections and RNA was pool-isolated with the RNA micro kit according to protocols given by the manufacturer (Qiagen, Hilden, Germany). cDNA synthesis and qRT-PCR were essentially performed as described [39].

### Reporter gene assays

Reporter gene assays in HEK293, CHO and PC12 cells [40] and primary cultured cortical neurons were performed as described previously [41] using a firefly luciferase reporter gene driven by the HSV-TK promoter and three clustered E-Box elements (CACGTG). CMV-driven expression plasmids coding for Flag-tagged mouse SHARP-1, HA-tagged rat SHARP-2, Flag-tagged mouse NPAS2, Flag-tagged mouse BMAL1, V5-tagged mouse PER1, V5-tagged mouse PER2, V5-tagged mouse CRY1 and V5-tagged mouse CRY2 were used (detailed information is available upon request). SHARP-1 and -2, NPAS2 and BMAL1 encoding constructs were assembled using Gateway-mediated recombination as described [41]. PER1,2 and CRY1,2 expression plasmids were kindly provided by Pablo Szendro, Max-Planck-Institute of experimental Endocrinology, Hannover, Germany.

### Quantitative RT-PCR

Quantitative PCR was performed with SybrGreen fluorescence assays analyzed with a ABI PRISM 7700 detection system (Perkin Elmer, Rodgau, Germany) as described [39]. The following primers directed against mouse transcripts were designed with Primer3 (http://biotools.umassmed.edu/bioapps/primer3_www.cgi) and used:

b-Actin-s: 5′- CTTCCTCCCTGGAGAAGAGC-3′
b-Actin-as: 5′- ATGCCACAGGATTCCATACC-3′
18S-rRNA-s: 5′- CTCAACACGGGAAACCTCAC-3′
18S-rRNA-as: 5′- CGCTCCACCAACTAAGAACG -3′
ATP5b-s: GGCACAATGCAGGAAAGG
ATP5b-as: TCAGCAGGCACATAGATAGCC
Per2-s: 5′-CAACACAGACGACAGCATCA-3′
Per2-as: 5′-TCCTGGTCCTCCTTCAACAC-3′
BMAL1-s: 5′- CATCCGCCATTAAAAGGAGA-3′
BMAL1-as: 5′- CGGAACAGTGTCTGGGATG-3′
NPAS2-s: 5′- CCCAGGAGTTACCAGTGCAG-3′
NPAS2-as: 5′- GAGGAATGCAGAGCAGTCG-3′
Per1-s: 5′- TCCTCCTCCTACACTGCCTCT-3′
Per1-as: 5′- TTGCTGACGACGGATCTTT-3′
c-Fos-s: 5′- GAATGGTGAAGACCGTGTCA-3′
c-Fos-as: 5′- TCTTCCTCTTCAGGAGATAGCTG-3′
c-Jun-s: 5′- CTGAGTGTGCGAGAGACAGC-3′
c-Jun-as: 5′- CCAAGTCCGTCCGTCTGT-3′

### Statistical Analysis

The behavioral data are presented as mean values ±SEM, qRT-PCR and Luciferase assays as means ±SD as indicated. Pairwise comparisons were evaluated by two tailed t-tests, repeated measures by two-way ANOVA with the factors time×genotype followed by Bonferroni post-hoc tests. A p value <0.05 was considered significant.

## Supporting Information

Figure S1 Characterization of the mutated Sharp-1 locus. (A) Schematic drawing of the Sharp-1 gene structure, CDS depicted as dark blue, 5′ and 3′ UTR in light blue. In the mutated Sharp-1 allele, exon3 (encoding the DNA binding domain) is replaced by a neomycin-resistance conferring cassette (neoR). RT-PCR primers located in exon 1 (Ex1s) and 4 (Ex4as) were used to detect aberrantly spliced transcripts, the primer pair amplifying exon 3 containing transcripts (Ex3s with Ex5as) was used to control for the absence of WT transcripts in the SHARP-1 mutant. (B) RT-PCR analysis with brain cDNA obtained from wild-type (+/+), Sharp-1 heterozygous (+/−) and homozygous (−/−) mutant mice. With the primer pairs Ex1s and Ex4as the expected 335 bp long product is amplified, in the hetero- and homozygous mutant additional aberrant products of increased size (>1.3 kB) are detected (left). With the primer pairs Ex3s and Ex5as, no product is amplified in homozygous mutants, cDNA input was checked with Gapdh primers (right). Three aberrantly PCR products (A, B, C) were detected in homozygous sharp-1 mutant cDNA (higher magnification depicted at the very right). (C) The PCR products A, B, C were subloned and sequenced. The analysis of the sequences revealed that transcripts corresponding to aberrant splice variant A may potentially result in an ORF of 49 aa lacking any known functional or homology region. Transcript A is not spliced at the junction from exon 2 to intron 2 and runs into a stop codon after seven residues. Transcripts B and C are unspliced already at exon 1 leading to an ORF of 21aa purely consisting of exon 1 encoded residues. (D) Putative aberrant protein variants from the mutated sharp-1 allele depicted as bar graphs. If expressed, Protein A (49 aa) will be comprised of exon 1 and 2 containing residues including a few additional aberrant amino acids, Protein B, C (21 aa) only contains the residues encoded by exon 1 followed by an immediate stop codon. (E) Reporter gene assay comparing the effect of the potentially expressed aberrant proteins A and B, C and wild-type SHARP-1 on an E-Box containing reporter. Whereas full-length SHARP-1 efficiently repressed reporter gene activation, neither constructs encoding aberrant products A or B, C showed any effect. RLUs = refererence light units. (Data represent mean values ±SD, n = 6 replicates).
(7.17 MB TIF)

Figure S2 Normal rhythmicity in LD and DD. Representative actograms showing wheel-running activities of wild-type (WT), Sharp-1 (S1−/−), Sharp-2 (S2−/−), and double null-mutant (S1/2−/−) mice in a 12 h light 12 h dark (LD) cycle and in complete darkness (DD). Lights-off phases are indicated by a grey, lights-on periods by a white background. Mice of all genotypes exhibit a robust circadian activity in LD, peaking during the first hours of darkness. In DD, rhythmic activity is retained with stepwise shifted wheel running onsets.
(0.59 MB TIF)

Figure S3 Light pulse induced phase shifting in WT and Sharp-2 mutant mice. (A,B) Representative actograms showing wheel-running activities of wild-type (WT) and Sharp-2 (S2−/−) mutant mice in DD and after a 15 min light pulse at CT14 (arrow, Aschoff type I protocol). (C) No significant differences in phase shift amplitudes were observed in S2−/− mice compared to the WT. However, a prolonged transition phase was seen in most S2−/− animals (red arrows in B) (Data represent mean values ±SEM, n = 12 per genotype).
(0.76 MB TIF)

Figure S4 Entrainment after rapid 8 h delayed LD cycle (experimental jet-lag). Representative actograms showing wheel-running activities of WT, S1−/−, S2−/−, S1−/−S2+−, S1+/−S2−/− and S1−/−S2−/− mutant mice exposed to an 8 h delayed LD cycle, respectively. Representative activity plots are depicted for each genotype; arrows indicate time of LD shift.
(0.47 MB TIF)

Figure S5 Repression of E-Box driven reporter gene. (A–C) Reporter gene assays with a firefly luciferase reporter construct driven by a herpes simplex thymidine-kinase (TK) minimal promoter and three upstream clustered CACGTG E-Box elements and BMAL1, NPAS2 and SHARP-1 or SHARP-2 expression plasmids performed in HEK293 cells (A), PC12 cells (B) and primary cultured mouse cortical neurons (C) as indicated. In all cell types, SHARP-1 and -2 significantly repress basal reporter gene activity irrespective of the co-transfection of BMAL1, NPAS2 or CLOCK encoding expression constructs. RLUs = refererence light units (Data represent mean values ±SD, n = 6 replicates).
(0.64 MB TIF)

Figure S6 Transcriptional repression in CHO cells. SHARP-1 and -2 repress basal and BMAL1/NPAS2 enhanced promoter activity in reporter gene assays performed in Chinese hamster ovary (CHO) cells. CHO cells were transfected with a luciferase reporter construct driven by a herpes simplex thymidine-kinase (TK) minimal promoter and three upstream clustered CACGTG E-Box elements and BMAL1, NPAS2 and SHARP-1 or SHARP-2 expression plasmids as indicated. In these cells, co-transfection of BMAL1 and NPAS2 encoding plasmids activate the reporter gene approximately 2.5- and 1.5-fold, respectively. When both BMAL1 and NPAS2 are co-transfected, reporter activity is increased more than 5-fold. Under all conditions, SHARP-1 represses reporter gene activity below the basal activity. SHARP-2 mediated repression appears to be less efficient in this context RLUs = refererence light units (Data represent mean values ±SD, n = 6 replicates).
(0.50 MB TIF)

Figure S7 SHARP-1 and -2 do not interact with BMAL1, NPAS2 or CLOCK bHLH domains. (A–C) Mammalian 2-hybrid analyses were performed in CHO cells to assess the potential interactions of the bHLH dimerization domains of SHARP-1 and -2 with BMAL1, NPAS2 or CLOCK. (A) BMAL1, NPAS2 or CLOCK were fused to the DNA binding domain of the yeast transcription factor Gal4 (referred to as Gal4) and respective expression constructs were co-transfected (B) with a Gal4 responsive luciferase reporter plasmid as well as with constructs encoding bHLH-domain fused to the strong transactivating domain from the Herpes Simplex virus (VP16) as indicated. (C) Whereas the bHLH domain fragment of BMAL1 interacts strongly with NPAS2 and CLOCK bHLH constructs, no interaction is detectable between SHARP-1/-2 and BMAL1, NPAS2 and CLOCK bHLH domain containing proteins. RLUs = refererence light units (Data represent mean values ±SD, n = 6 replicates).
(0.50 MB TIF)

Figure S8 BMAL1 but not SHARP-1 or SHARP-2 can re-localize EYFP-NPAS2 to the nucleus. (A–C) Assessment of the cytoplasm-nucleus localization of EYFP-NPAS2 by BMAL1 and SHARP-1 or -2 in COS1 cells. (A) Domain structures of the expression constructs. EYFP-NPAS2, the enhanced yellow fluorescent protein (EYFP) is fused N-terminally to full-length NPAS2 that contains a bHLH and two PAS domains. BMAL1, full-length BMAL1 also harbours a bHLH and two PAS domains. SHARP-1 and SHARP-2, full length SHARP-1 and -2 encoding constructs including the bHLH, orange and C-terminal (CTD) domains. (B) Representative microscopic pictures depicting the EYFP fluorescence of EYFP-NPAS2 when transfected separately or in combination with BMAL1, SHARP-1 or SHARP-2 encoding constructs in COS1 cells. (C) Quantitative analyses of the subcellular localizations (white bars, nucleus; grey bars, both nucleus and cytoplasm (N/C); black bars, cytoplasm) of EYFP-NPAS2. Whereas BMAL1 efficiently alters the EYFP-NPAS2 fluorescence from an almost exclusive cytoplasmic to a nuclear localization, SHARP-1 and -2 do not show any effect in this assay. Data are given as percentage of means, n = 4 with more than 20 microscopic fields (each with at least 3–4 cells) analyzed. Scale bar = 50 µm.
(2.66 MB TIF)

Figure S9 SHARP-1 and -2 are E-Box-independent transcriptional co-activators of BMAL1/NPAS2 or BMAL1/CLOCK. (A–C) Reporter gene assays monitoring the transcriptional effect of SHARP-1 and -2 using an E-Box independent assay in CHO cells. (A) Schematic domain structure of constructs used. Gal4-BMAL1, the DNA binding domain of Gal4 was fused N-terminally to full-length BMAL1. NPAS2 and CLOCK, full-length NPAS2 and CLOCK expression constructs, respectively. VP16-NPAS2 and VP16-CLOCK, the strong transactivation domain VP16 was fused N-terminally to full-length NPAS2 and CLOCK expression constructs, respectively. SHARP-1 and SHARP-2, expression constructs encoding full length SHARP-1 and -2, respectively. (B) Schematic drawing of the assay principle. Gal4-BMAL1/NPAS2 or CLOCK complexes are monitored with a Gal4-dependent reporter plasmid (G5-Luci) in the absence and presence of SHARP-1 or SHARP-2. (C) Gal4-BMAL1 activates along with exogenous NPAS2 or CLOCK basal reporter gene activity more than 8-fold above basal promoter activity (small inset figure). Additional co-transfection with SHARP-1 and SHARP-2 strongly elevates reporter gene transcription. SHARP-1 further stimulates Gal4-BMAL1/VP16-NPAS2 and VP16-CLOCK activities strongly, whereas SHARP-2 co-activation is less pronounced. RLUs = refererence light units (Data represent mean values ±SD, n = 6 replicates).
(0.61 MB TIF)
