# Supplementary figures and images for: Disturbed Clockwork Resetting in Sharp-1 and Sharp-2 Single and Double Mutant Mice
Source: PLoS One. 2008 Jul 23;3(7):e2762. doi: 10.1371/journal.pone.0002762 (PMC2447179; doi:10.1371/journal.pone.0002762)

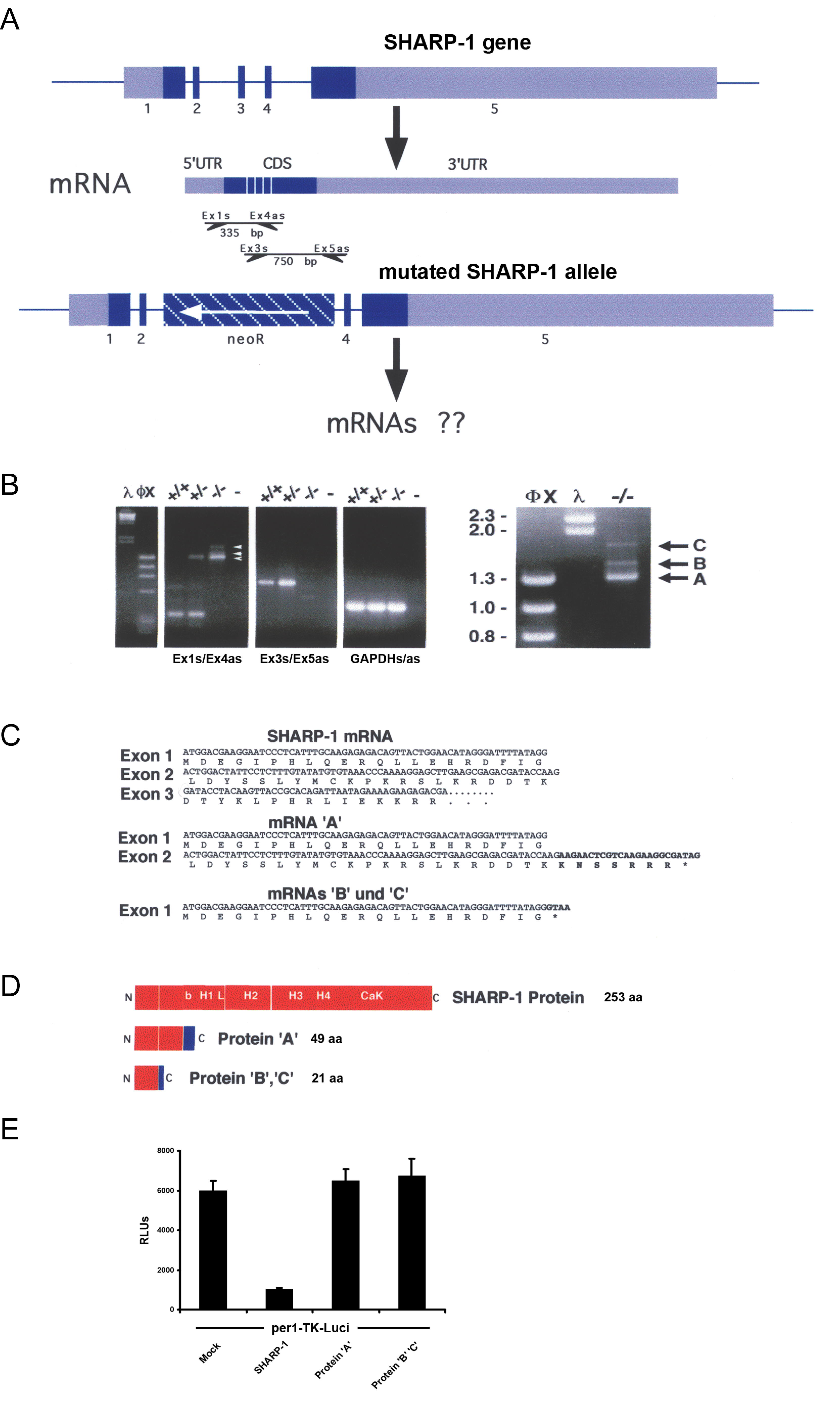

Supplement: Figure S1 — Characterization of the mutated Sharp-1 locus. (A) Schematic drawing of the Sharp-1 gene structure, CDS depicted as dark blue, 5′ and 3′ UTR in light blue. In the mutated Sharp-1 allele, exon3 (encoding the DNA binding domain) is replaced by a neomycin-resistance conferring cassette (neoR). RT-PCR primers located in exon 1 (Ex1s) and 4 (Ex4as) were used to detect aberrantly spliced transcripts, the primer pair amplifying exon 3 containing transcripts (Ex3s with Ex5as) was used to control for the absence of WT transcripts in the SHARP-1 mutant. (B) RT-PCR analysis with brain cDNA obtained from wild-type (+/+), Sharp-1 heterozygous (+/−) and homozygous (−/−) mutant mice. With the primer pairs Ex1s and Ex4as the expected 335 bp long product is amplified, in the hetero- and homozygous mutant additional aberrant products of increased size (>1.3 kB) are detected (left). With the primer pairs Ex3s and Ex5as, no product is amplified in homozygous mutants, cDNA input was checked with Gapdh primers (right). Three aberrantly PCR products (A, B, C) were detected in homozygous sharp-1 mutant cDNA (higher magnification depicted at the very right). (C) The PCR products A, B, C were subloned and sequenced. The analysis of the sequences revealed that transcripts corresponding to aberrant splice variant A may potentially result in an ORF of 49 aa lacking any known functional or homology region. Transcript A is not spliced at the junction from exon 2 to intron 2 and runs into a stop codon after seven residues. Transcripts B and C are unspliced already at exon 1 leading to an ORF of 21aa purely consisting of exon 1 encoded residues. (D) Putative aberrant protein variants from the mutated sharp-1 allele depicted as bar graphs. If expressed, Protein A (49 aa) will be comprised of exon 1 and 2 containing residues including a few additional aberrant amino acids, Protein B, C (21 aa) only contains the residues encoded by exon 1 followed by an immediate stop codon. (E) Reporter gene assay co [file pone.0002762.s001.tif]

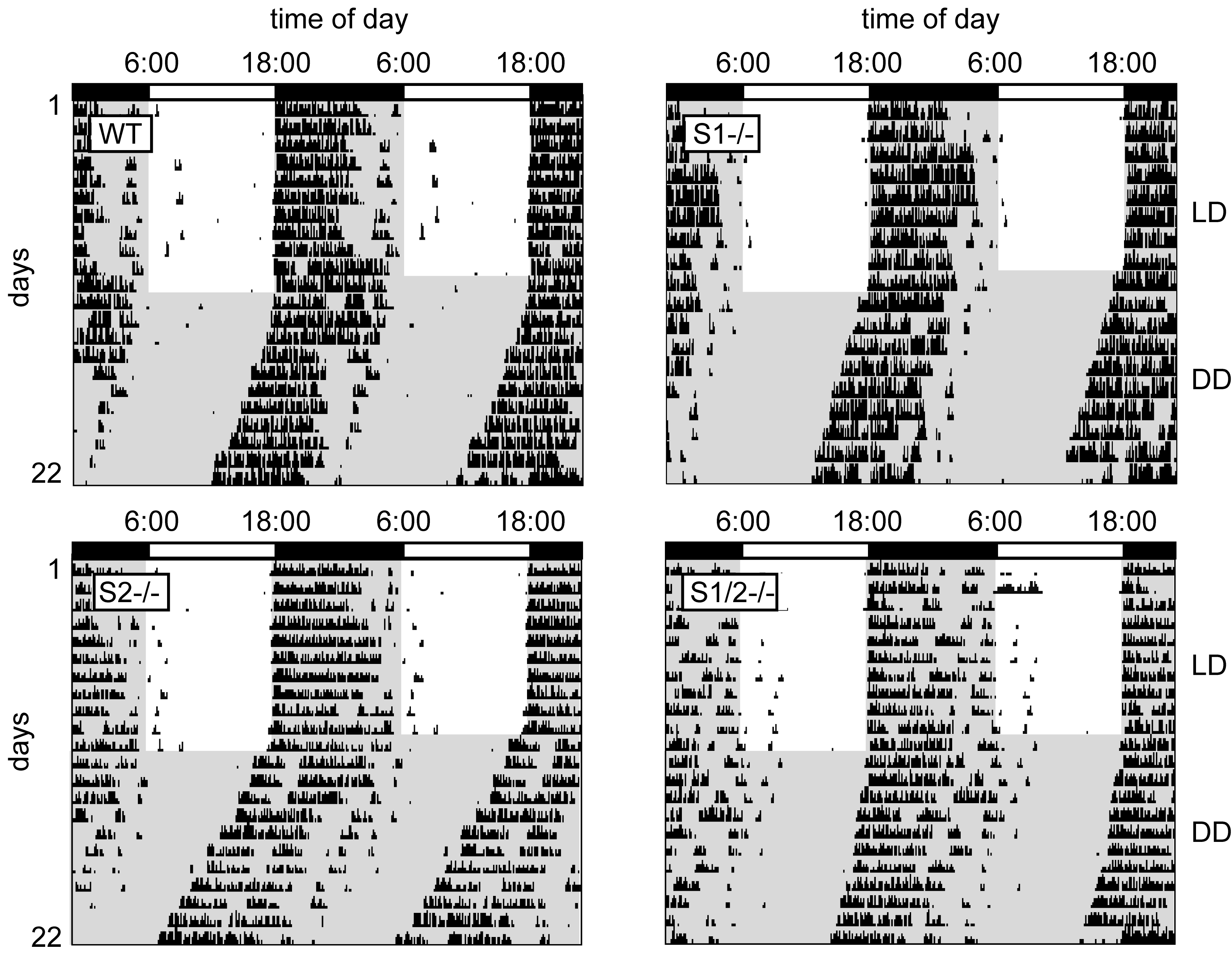

Supplement: Figure S2 — Normal rhythmicity in LD and DD. Representative actograms showing wheel-running activities of wild-type (WT), Sharp-1 (S1−/−), Sharp-2 (S2−/−), and double null-mutant (S1/2−/−) mice in a 12 h light 12 h dark (LD) cycle and in complete darkness (DD). Lights-off phases are indicated by a grey, lights-on periods by a white background. Mice of all genotypes exhibit a robust circadian activity in LD, peaking during the first hours of darkness. In DD, rhythmic activity is retained with stepwise shifted wheel running onsets. (0.59 MB TIF) [file pone.0002762.s002.tif]

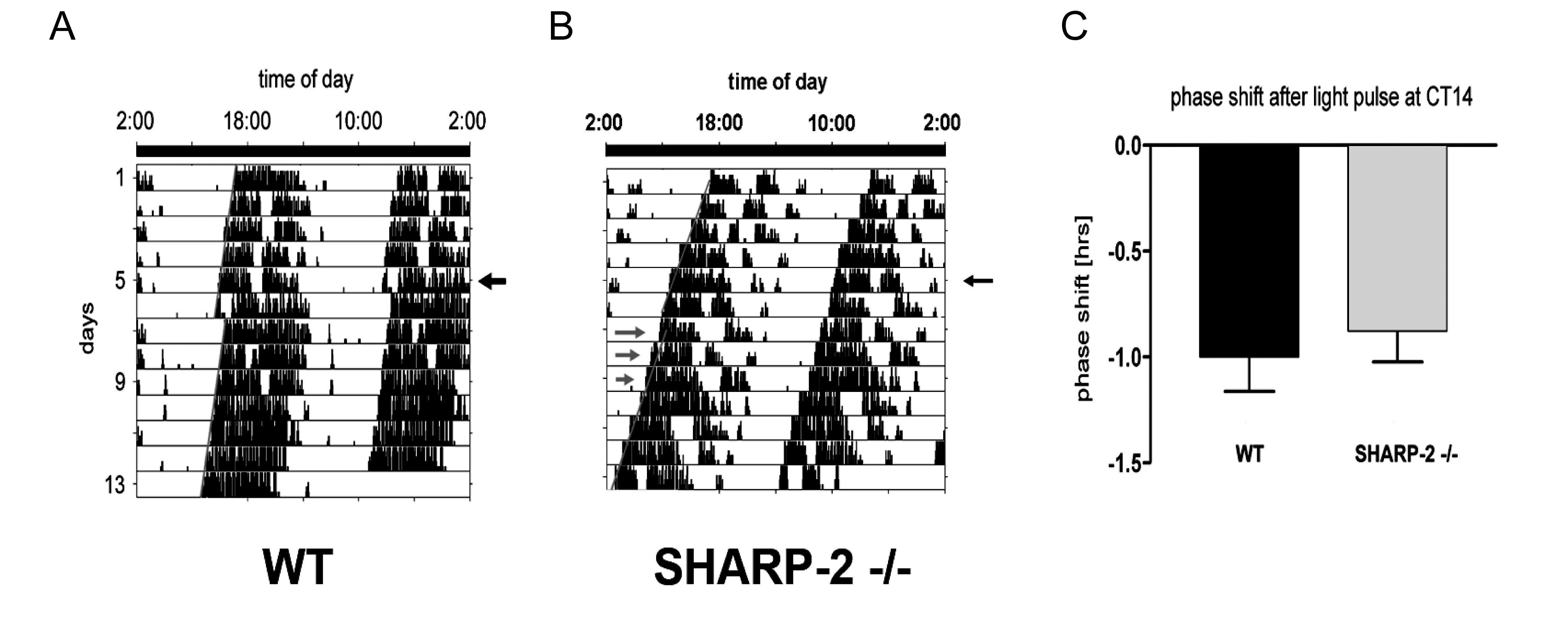

Supplement: Figure S3 — Light pulse induced phase shifting in WT and Sharp-2 mutant mice. (A,B) Representative actograms showing wheel-running activities of wild-type (WT) and Sharp-2 (S2−/−) mutant mice in DD and after a 15 min light pulse at CT14 (arrow, Aschoff type I protocol). (C) No significant differences in phase shift amplitudes were observed in S2−/− mice compared to the WT. However, a prolonged transition phase was seen in most S2−/− animals (red arrows in B) (Data represent mean values ±SEM, n = 12 per genotype). (0.76 MB TIF) [file pone.0002762.s003.tif]

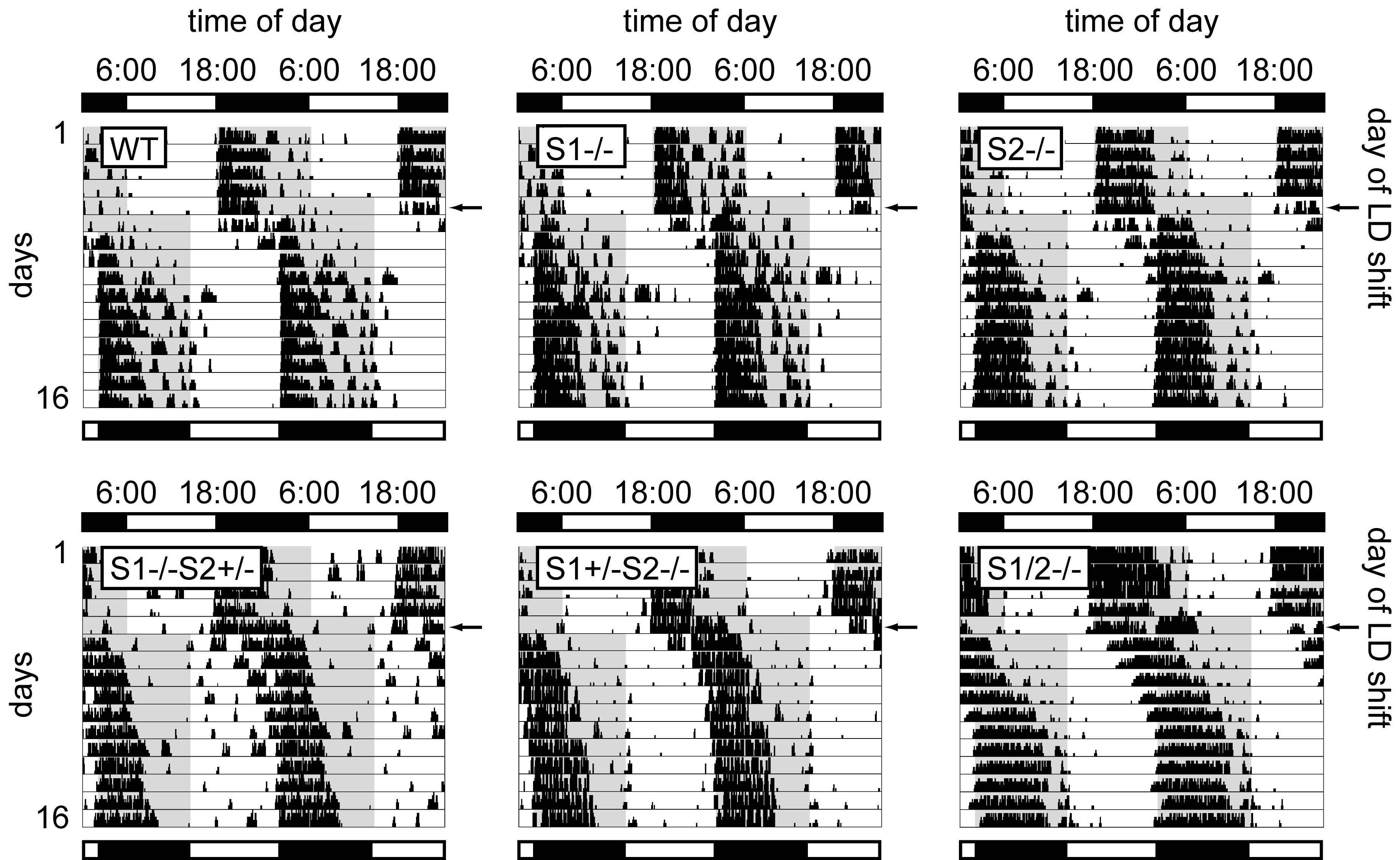

Supplement: Figure S4 — Entrainment after rapid 8 h delayed LD cycle (experimental jet-lag). Representative actograms showing wheel-running activities of WT, S1−/−, S2−/−, S1−/−S2+−, S1+/−S2−/− and S1−/−S2−/− mutant mice exposed to an 8 h delayed LD cycle, respectively. Representative activity plots are depicted for each genotype; arrows indicate time of LD shift. (0.47 MB TIF) [file pone.0002762.s004.tif]

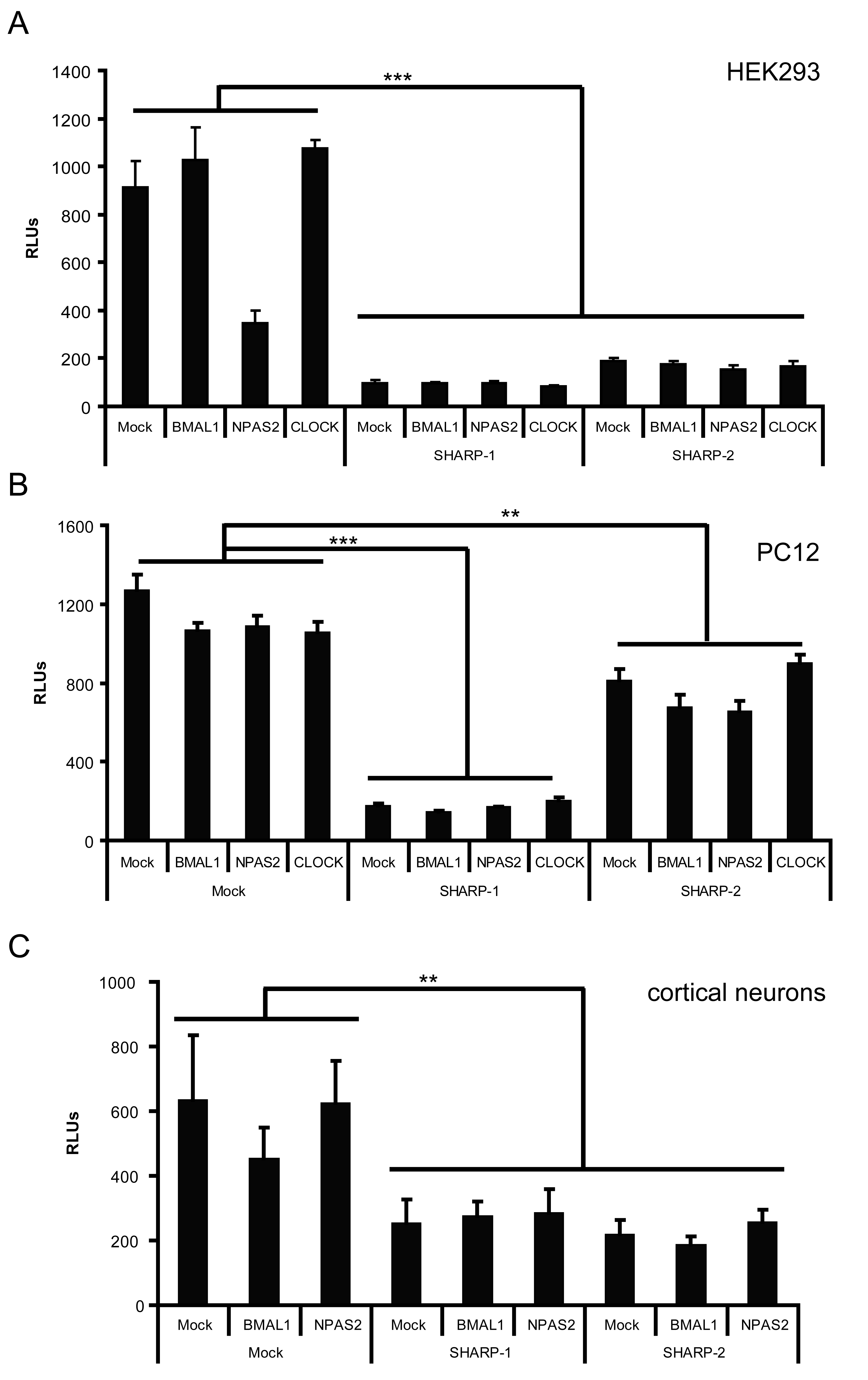

Supplement: Figure S5 — Repression of E-Box driven reporter gene. (A–C) Reporter gene assays with a firefly luciferase reporter construct driven by a herpes simplex thymidine-kinase (TK) minimal promoter and three upstream clustered CACGTG E-Box elements and BMAL1, NPAS2 and SHARP-1 or SHARP-2 expression plasmids performed in HEK293 cells (A), PC12 cells (B) and primary cultured mouse cortical neurons (C) as indicated. In all cell types, SHARP-1 and -2 significantly repress basal reporter gene activity irrespective of the co-transfection of BMAL1, NPAS2 or CLOCK encoding expression constructs. RLUs = refererence light units (Data represent mean values ±SD, n = 6 replicates). (0.64 MB TIF) [file pone.0002762.s005.tif]

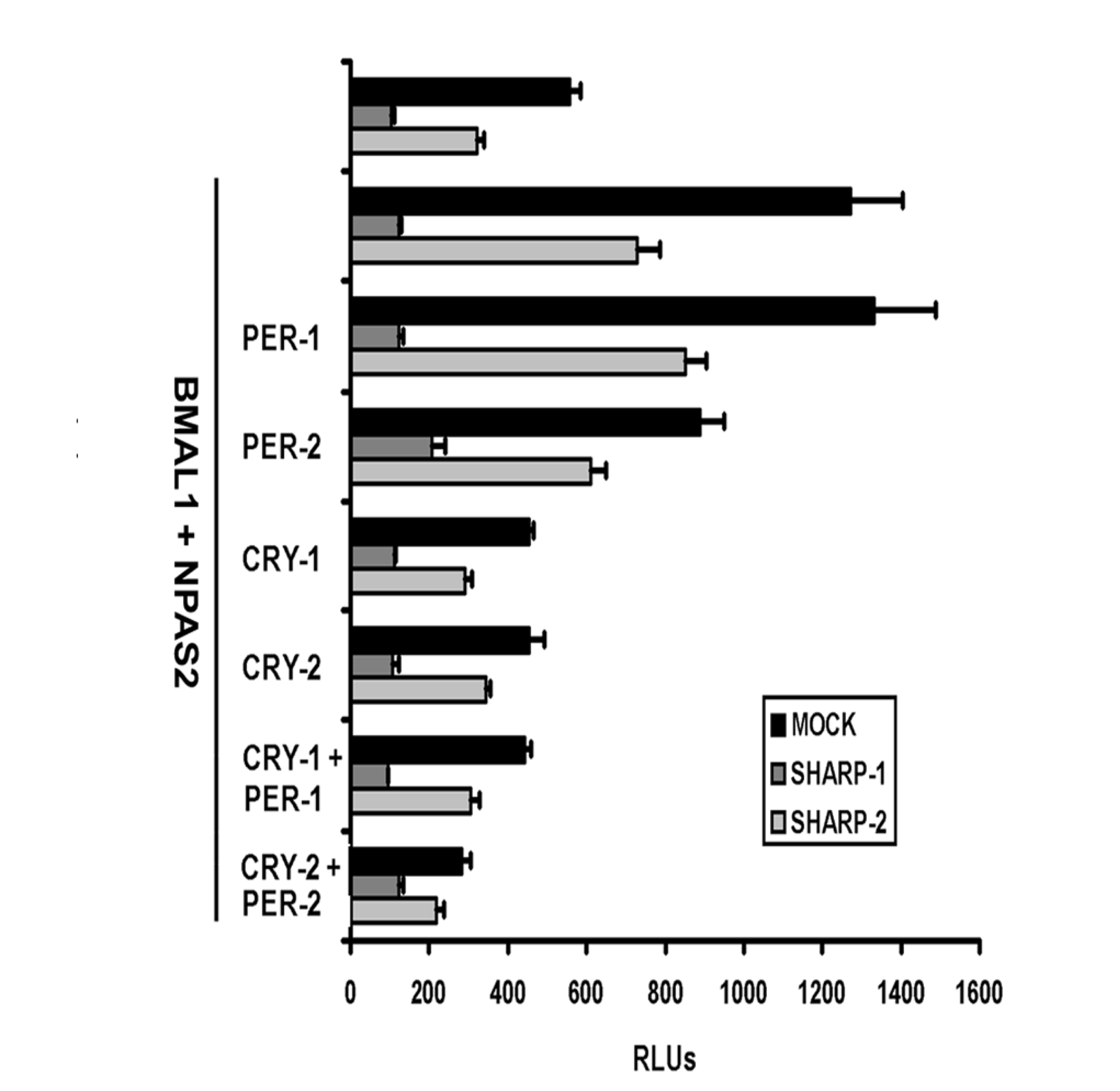

Supplement: Figure S6 — Transcriptional repression in CHO cells. SHARP-1 and -2 repress basal and BMAL1/NPAS2 enhanced promoter activity in reporter gene assays performed in Chinese hamster ovary (CHO) cells. CHO cells were transfected with a luciferase reporter construct driven by a herpes simplex thymidine-kinase (TK) minimal promoter and three upstream clustered CACGTG E-Box elements and BMAL1, NPAS2 and SHARP-1 or SHARP-2 expression plasmids as indicated. In these cells, co-transfection of BMAL1 and NPAS2 encoding plasmids activate the reporter gene approximately 2.5- and 1.5-fold, respectively. When both BMAL1 and NPAS2 are co-transfected, reporter activity is increased more than 5-fold. Under all conditions, SHARP-1 represses reporter gene activity below the basal activity. SHARP-2 mediated repression appears to be less efficient in this context RLUs = refererence light units (Data represent mean values ±SD, n = 6 replicates). (0.50 MB TIF) [file pone.0002762.s006.tif]

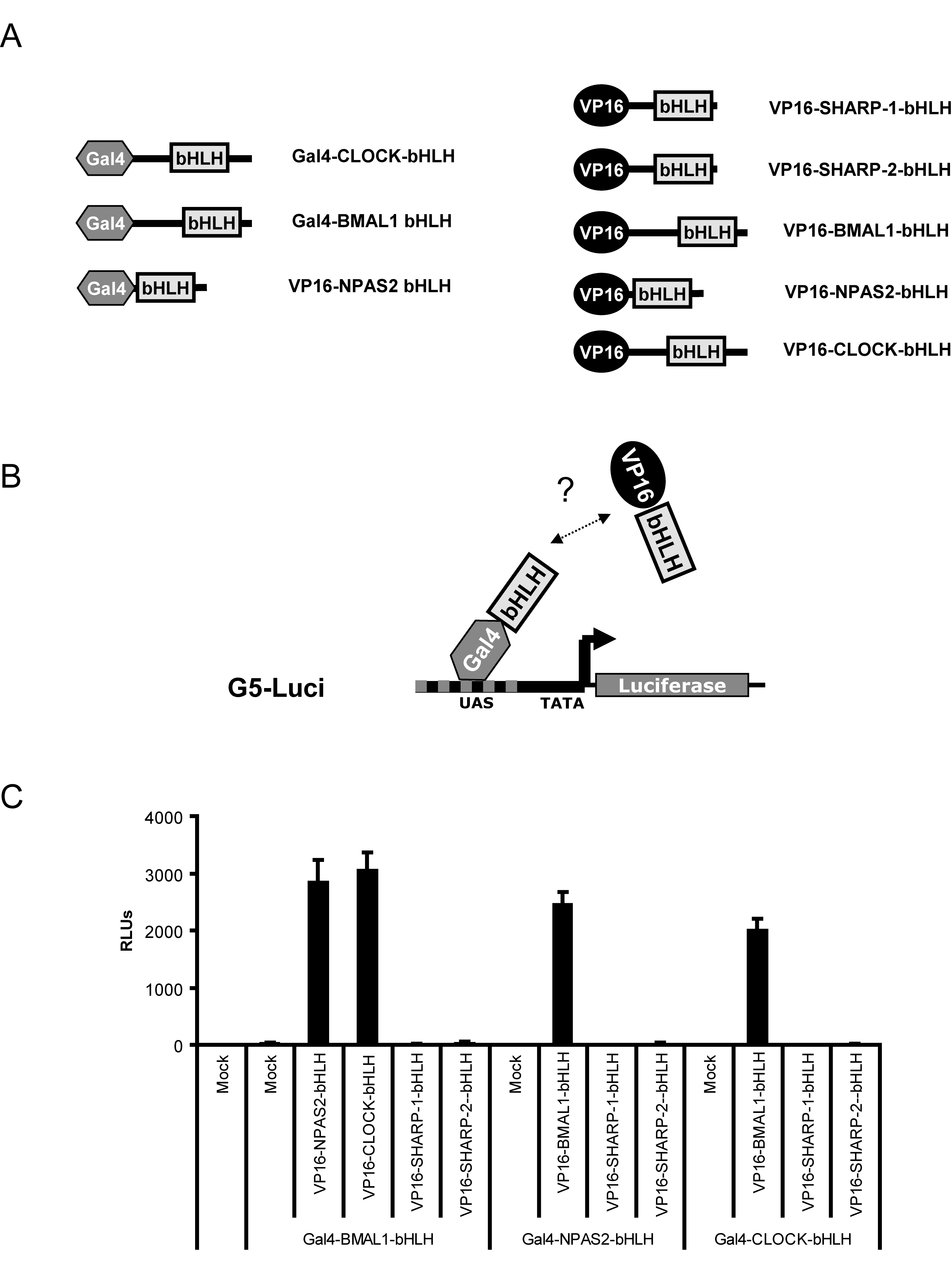

Supplement: Figure S7 — SHARP-1 and -2 do not interact with BMAL1, NPAS2 or CLOCK bHLH domains. (A–C) Mammalian 2-hybrid analyses were performed in CHO cells to assess the potential interactions of the bHLH dimerization domains of SHARP-1 and -2 with BMAL1, NPAS2 or CLOCK. (A) BMAL1, NPAS2 or CLOCK were fused to the DNA binding domain of the yeast transcription factor Gal4 (referred to as Gal4) and respective expression constructs were co-transfected (B) with a Gal4 responsive luciferase reporter plasmid as well as with constructs encoding bHLH-domain fused to the strong transactivating domain from the Herpes Simplex virus (VP16) as indicated. (C) Whereas the bHLH domain fragment of BMAL1 interacts strongly with NPAS2 and CLOCK bHLH constructs, no interaction is detectable between SHARP-1/-2 and BMAL1, NPAS2 and CLOCK bHLH domain containing proteins. RLUs = refererence light units (Data represent mean values ±SD, n = 6 replicates). (0.50 MB TIF) [file pone.0002762.s007.tif]

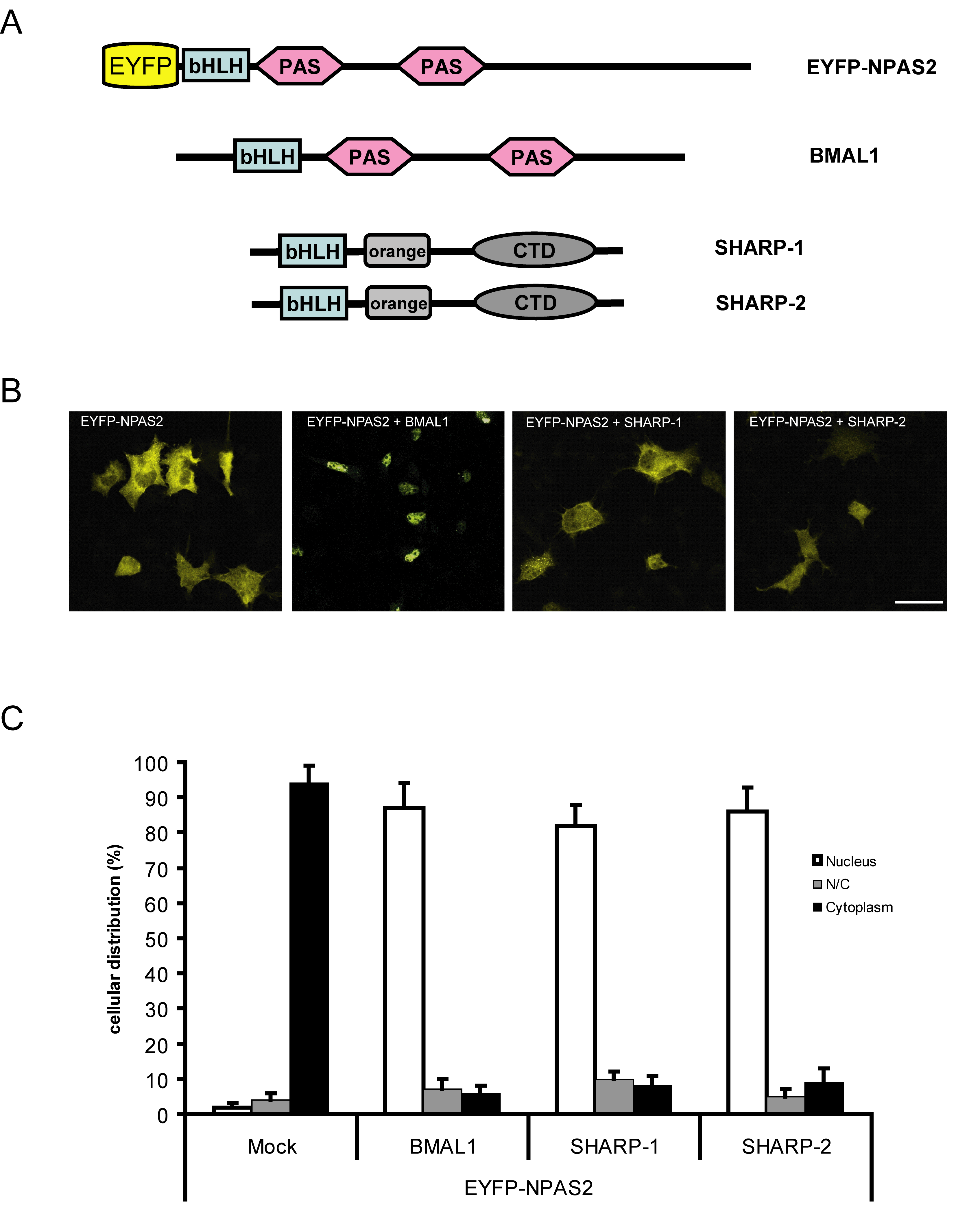

Supplement: Figure S8 — BMAL1 but not SHARP-1 or SHARP-2 can re-localize EYFP-NPAS2 to the nucleus. (A–C) Assessment of the cytoplasm-nucleus localization of EYFP-NPAS2 by BMAL1 and SHARP-1 or -2 in COS1 cells. (A) Domain structures of the expression constructs. EYFP-NPAS2, the enhanced yellow fluorescent protein (EYFP) is fused N-terminally to full-length NPAS2 that contains a bHLH and two PAS domains. BMAL1, full-length BMAL1 also harbours a bHLH and two PAS domains. SHARP-1 and SHARP-2, full length SHARP-1 and -2 encoding constructs including the bHLH, orange and C-terminal (CTD) domains. (B) Representative microscopic pictures depicting the EYFP fluorescence of EYFP-NPAS2 when transfected separately or in combination with BMAL1, SHARP-1 or SHARP-2 encoding constructs in COS1 cells. (C) Quantitative analyses of the subcellular localizations (white bars, nucleus; grey bars, both nucleus and cytoplasm (N/C); black bars, cytoplasm) of EYFP-NPAS2. Whereas BMAL1 efficiently alters the EYFP-NPAS2 fluorescence from an almost exclusive cytoplasmic to a nuclear localization, SHARP-1 and -2 do not show any effect in this assay. Data are given as percentage of means, n = 4 with more than 20 microscopic fields (each with at least 3–4 cells) analyzed. Scale bar = 50 µm. (2.66 MB TIF) [file pone.0002762.s008.tif]

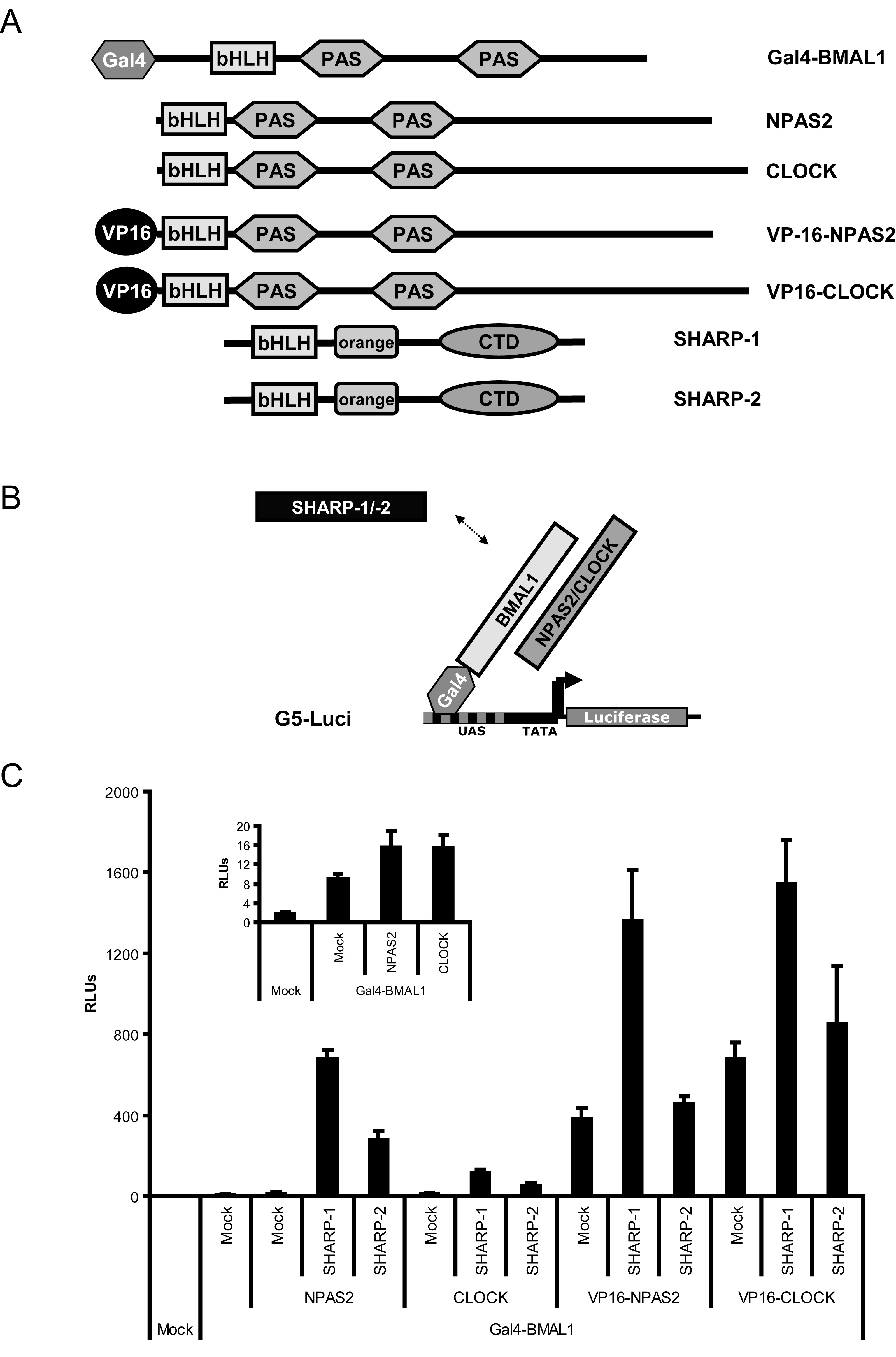

Supplement: Figure S9 — SHARP-1 and -2 are E-Box-independent transcriptional co-activators of BMAL1/NPAS2 or BMAL1/CLOCK. (A–C) Reporter gene assays monitoring the transcriptional effect of SHARP-1 and -2 using an E-Box independent assay in CHO cells. (A) Schematic domain structure of constructs used. Gal4-BMAL1, the DNA binding domain of Gal4 was fused N-terminally to full-length BMAL1. NPAS2 and CLOCK, full-length NPAS2 and CLOCK expression constructs, respectively. VP16-NPAS2 and VP16-CLOCK, the strong transactivation domain VP16 was fused N-terminally to full-length NPAS2 and CLOCK expression constructs, respectively. SHARP-1 and SHARP-2, expression constructs encoding full length SHARP-1 and -2, respectively. (B) Schematic drawing of the assay principle. Gal4-BMAL1/NPAS2 or CLOCK complexes are monitored with a Gal4-dependent reporter plasmid (G5-Luci) in the absence and presence of SHARP-1 or SHARP-2. (C) Gal4-BMAL1 activates along with exogenous NPAS2 or CLOCK basal reporter gene activity more than 8-fold above basal promoter activity (small inset figure). Additional co-transfection with SHARP-1 and SHARP-2 strongly elevates reporter gene transcription. SHARP-1 further stimulates Gal4-BMAL1/VP16-NPAS2 and VP16-CLOCK activities strongly, whereas SHARP-2 co-activation is less pronounced. RLUs = refererence light units (Data represent mean values ±SD, n = 6 replicates). (0.61 MB TIF) [file pone.0002762.s009.tif]
